# Perioperative Outcomes of Proximal and Distal Gastric Bypass in Patients with BMI Ranged 50–60 kg/m^2^—A Double-Blind, Randomized Controlled Trial

**DOI:** 10.1007/s11695-015-1621-y

**Published:** 2015-03-12

**Authors:** Marius Svanevik, Hilde Risstad, Dag Hofsø, Carl Fredrik Schou, Brita Solheim, Torgeir T. Søvik, Jon Kristinsson, Jøran Hjelmesæth, Tom Mala, Rune Sandbu

**Affiliations:** 1 Morbid Obesity Center, Vestfold Hospital Trust, Halfdan Wilhelmsens alle 17, 3103 Tønsberg, Norway; 2 Institute of Clinical Medicine, University of Oslo, Oslo, Norway; 3 Department of Morbid Obesity and Bariatric Surgery, Oslo University Hospital, Oslo, Norway; 4 Department of Gastrointestinal Surgery, Oslo University Hospital, Oslo, Norway

**Keywords:** Bariatric surgery, Gastric bypass, Malabsorptive gastric bypass, Distal gastric bypass, Malabsorptive procedure, Laparoscopic bariatric surgery, Randomized controlled trial

## Abstract

**Background:**

Proximal Roux-en-Y gastric bypass may not ensure adequate weight loss in superobese patients. Bypassing a longer segment of the small bowel may increase weight loss. The objective of the study was to compare the perioperative outcomes of laparoscopic proximal and distal gastric bypass in a double-blind randomized controlled trial of superobese patients. The study was conducted at two public tertiary care obesity centers in Norway.

**Methods:**

Patients with body mass index (BMI) 50–60 kg/m^2^ were randomly assigned to a proximal (150 cm alimentary limb) or a distal (150 cm common channel) gastric bypass. The biliopancreatic limb was 50 cm in both operations. Patients and follow-up personnel were blinded to the type of procedure. Thirty-day outcomes including complications are reported.

**Results:**

We operated on 115 patients, of whom two were excluded at surgery, leaving 56 and 57 patients in the proximal group and distal group, respectively. The median (range) operating time was 72 (36–151) and 101 (59–227) min, respectively (*p* < 0.001). Two distal procedures were converted to laparotomy during the primary procedure. Median length of hospital stay was 2 (1–4) days in the proximal group and 2 (1–24) days in the distal group. The number of patients with complications and complications categorized according to the Contracted Accordion classification did not differ significantly. However, all six reoperations were performed in the distal group, of which three were completed by laparoscopy (*p* = 0.01 between groups). There were no deaths.

**Conclusions:**

In superobese patients with BMI between 50 and 60 kg/m^2^, distal gastric bypass was associated with longer operating time and more severe complications resulting in reoperation than proximal gastric bypass.

## Introduction

The mechanisms of weight loss after bariatric surgery include restriction of food intake and malabsorption of nutrients. For some procedures, altered gastrointestinal endocrine physiology may mediate weight loss through modulation of appetite and energy homeostasis [1, 2].

The Roux-en-Y gastric bypass provides substantial and sustained weight loss in morbidly obese patients [3, 4]. Superobese patients (body mass index, BMI ≥ 50 kg/m^2^), however, may remain morbidly obese after this procedure [5, 6]. The biliopancreatic diversion with duodenal switch induces greater weight loss than proximal gastric bypass, possibly through greater malabsorption, but is seldom performed as a primary bariatric procedure. The biliopancreatic diversion is a more challenging and technical procedure to perform and associated with a high frequency of short- and long-term adverse events including protein-calorie malnutrition [7, 8].

There is no general consensus as to the optimal intestinal limb lengths to be used in gastric bypass for superobese patients [9]. Lengthening the alimentary or the biliopancreatic limb or shortening the common channel may increase malabsorption and subsequent weight loss [10]. Brolin et al. introduced the distal gastric bypass with a common channel of 75 cm, resulting in increased weight loss but also severe metabolic problems [11]. Similar problems with malnutrition and liver failure occurred when combining a longer common channel of 150 cm with a short alimentary limb [12]. Building upon the work of Nelson [13], we hypothesize that a common channel of 150 cm combined with a long alimentary limb may ensure malabsorption without severe metabolic problems.

To the best of our knowledge, no randomized controlled trial has compared weight loss outcomes or complications of proximal and distal gastric bypass in superobese patients. In this first report, we compare the perioperative outcomes of the two procedures.

## Methods

### Study Design and Setting

This study is part of an ongoing two-center randomized (1:1) double-blind, controlled trial comparing proximal and distal gastric bypass in superobese patients. The trial was approved by the Regional Ethics Committee and is registered in Clinicaltrials.gov, identifier NCT00821197. Signed informed consent was obtained from all patients.

The eligibility criteria for trial inclusion were BMI 50 to 60 kg/m^2^ at the time of referral (48 to 62 kg/m^2^ at enrollment) and age between 20 and 60 years. Exclusion criteria included previous bariatric or major abdominal surgery, urolithiasis, chronic liver disease, and conditions associated with poor compliance. All patients referred to bariatric surgery at the study centers were reviewed for inclusion in the period January 2011 to March 2013. The patients were operated on between March 2011 and April 2013.

Both participating hospitals, Oslo University Hospital and Vestfold Hospital Trust, are public tertiary care centers, each performing over 200 bariatric procedures annually. Prior to the study, the centers had performed over 1000 proximal gastric bypass procedures and a total of 40 distal gastric bypasses. All procedures were performed by senior surgeons at Vestfold Hospital Trust (RS and BS) and Oslo University Hospital (CFS, JK, and TM) in order to reduce potential learning curve effects. All senior surgeons had performed 200 or more bariatric procedures, mostly gastric bypass, prior to the start of the study.

## Study Treatment

All enrolled patients followed standard preoperative logistics, including schooling on nutrition, exercise, and what to expect after surgery. All patients were prescribed a low-calorie diet (<1000 kcal/day) 3 weeks before surgery and recommended a preoperative weight loss of 5–10 %.

At each study visit, all patients underwent a physical examination and data including demographics, comorbidities, and medications were registered. Fasting blood samples were obtained, and study questionnaires completed at the hospital. Weight was measured with participants wearing light clothing and no shoes.

Type 2 diabetes was defined as fasting serum glucose ≥7.0 mmol/L, glycated hemoglobin (HbA1c) ≥6.5 %, or the use of at least one glucose-lowering medication [14]. Hypertension was defined as blood pressure ≥140/90 mmHg or the use of at least one antihypertensive medication [15].

## Surgical Procedures

Both procedures were performed with an antegastric antecolic Roux-en-Y configuration using linear staplers and an omega loop [16]. Standard port placement was applied with four bladeless trocars and a Nathanson liver retractor (Cook Medical), and an extra 5-mm port was inserted if needed. All stapling was performed using a linear stapler, with blue cartridges for the stomach and white cartridges for the small bowel. The pouch was created by stapling the stomach horizontally from the minor curvature and vertically to create a gastric pouch of about 25 ml. The gastrojejunostomy was created using a 45-mm stapler with blue cartridge and completed with a running suture. The omentum was not transected routinely. The biliopancreatic limb was 50 cm in both procedures. Limb lengths were measured sequentially using 5-cm markers on the graspers; the bowel was held taut but not stretched.

**Fig. 1:**
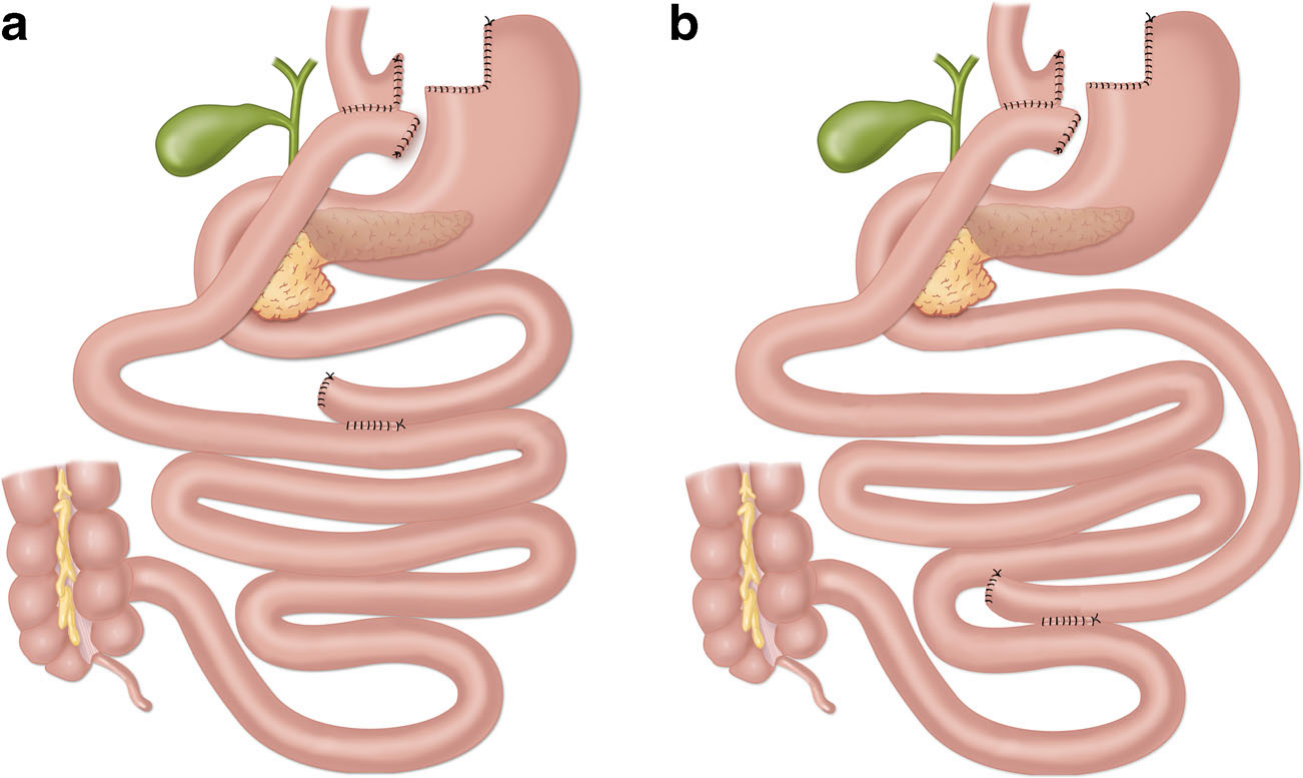
Anatomical differences between **a** proximal gastric bypass and **b** distal gastric bypass

Following the creation of the gastrojejunostomy, the alimentary limb was measured to 150 cm from the gastrojejunostomy in preparation for a proximal gastric bypass, with a side-to-side jejunojejunostomy created using a 45-mm stapler cartridge and closed with a running suture. In preparation for a distal gastric bypass, the common channel was measured to 150 cm from the ileocecal junction and marked with a suture. The bowel was then run from the gastric pouch until reaching the marker, and a side-to-side jejunoileostomy was created using a 45-mm stapler cartridge and closed with a running suture. The Roux-en-Y configuration was completed by dividing the jejunum between the anastomoses, with the patency of the gastrojejunostomy evaluated by instilling diluted methylene blue in a nasogastric tube. The fascial defects after trocars were not closed. See Fig. 1 for illustration.

## Postoperative Care and Follow-Up

Participants were advised to consume liquids only during the first postoperative week, to adhere to a semiliquid diet during the second week, and to gradually introduce normal food to their diet during the third week after surgery. Low molecular weight heparin was administered subcutaneously the first 10 days after surgery, and all patients were recommended a standard daily oral supplement of a multivitamin and mineral tablet, iron, calcium, and vitamin D. Vitamin B12 was administered through injections, and ursodeoxycholic acid prescribed for 6 months in order to prevent gallstone formation, with the exception of patients who had previously undergone cholecystectomy. The first scheduled follow-up was at 6 weeks after surgery in the outpatient clinic, where we also registered 30-day complications.

## Study Endpoints

In this report, we present perioperative outcomes to evaluate short-term safety. The primary endpoint of the overall study is change in BMI 2 years after surgery. Other secondary endpoints include body composition, anthropometry, obesity-related comorbidities, health-related quality of life, gastrointestinal symptoms, and adverse events including nutritional deficiencies. All patients will be followed for 5 years.

## Classification of Complications

We used the Contracted Accordion Classification to assess 30-day complications. The severity of complications is categorized from 1 to 4 according to the therapy required to correct the event [17].

## Sample Size and Randomization

To estimate sample size in the study, we reviewed weight outcome in morbidly obese patients who had gastric bypass surgery at the study centers. These patients had a mean BMI reduction of 15.8 kg/m^2^ (SD 4.7) 1 year after surgery (unpublished data). We hypothesized that a distal gastric bypass would be associated with a further BMI reduction of 3.0 kg/m^2^ 1 year after surgery, i.e., an estimated 1-year change in BMI of 18.8 kg/m^2^ (SD 5.0). Assuming a similar change in BMI within the groups at 2 years and 80 % power to detect a significant (*p* < 0.05) difference between the groups, we calculated that 88 patients would have to complete the study. We decided to enroll a minimum of 112 participants in order to allow for possible dropouts. The participants were block randomized (random blocks of 4 and 6) using computer-based randomization and sealed envelopes to conceal allocation.

Study participants and follow-up personnel were blinded for type of procedure. The operating surgeons were not involved in the study follow-up except in cases of surgical emergencies. Information about type of procedure was available for medical emergencies. The blinding will be lifted 5 years after surgery.

## Statistical Methods

Normally distributed values are reported as mean (SD) while non-normally distributed values are reported as median (range). Chi-square and Fisher’s exact test were used to compare proportions. Student’s *t* test or Mann–Whitney *U* test were applied for the analysis of continuous data as appropriate. Ordered categorical data was compared between the treatment groups with the Mann–Whitney *U* test. A two-sided *p* value < 0.05 was considered statistically significant. Statistical analyses were performed using SPSS for Windows, version 16.0, Chicago, SPSS Inc.

## Results

**Fig. 2:**
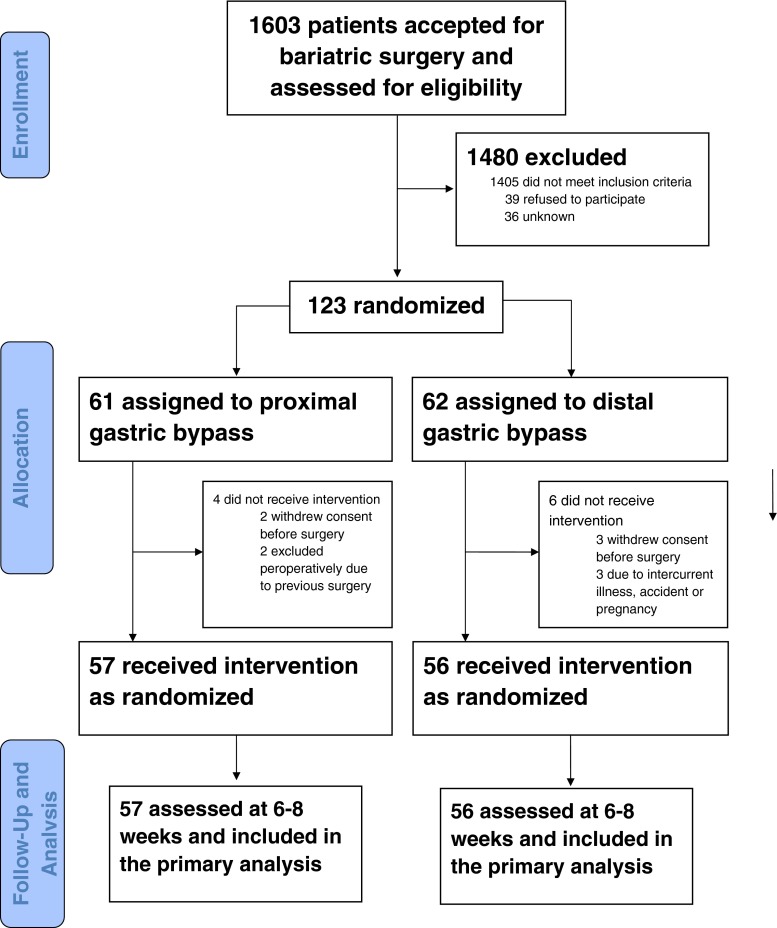
CONSORT flow chart

A total of 123 patients were eligible for study inclusion and randomized to proximal or distal gastric bypass (Fig. 2). Eight patients were later either excluded or withdrew for various reasons, while two patients randomized to proximal gastric bypass were excluded during surgery when it was discovered that they had undergone major abdominal surgery. One patient underwent laparoscopic sleeve gastrectomy, the other proximal gastric bypass, with neither included in the study follow-up. A total of 112 patients met up at the first scheduled follow-up while one was contacted by phone, with all patients included in the analyses (Fig. 2).

**Fig. 3:**
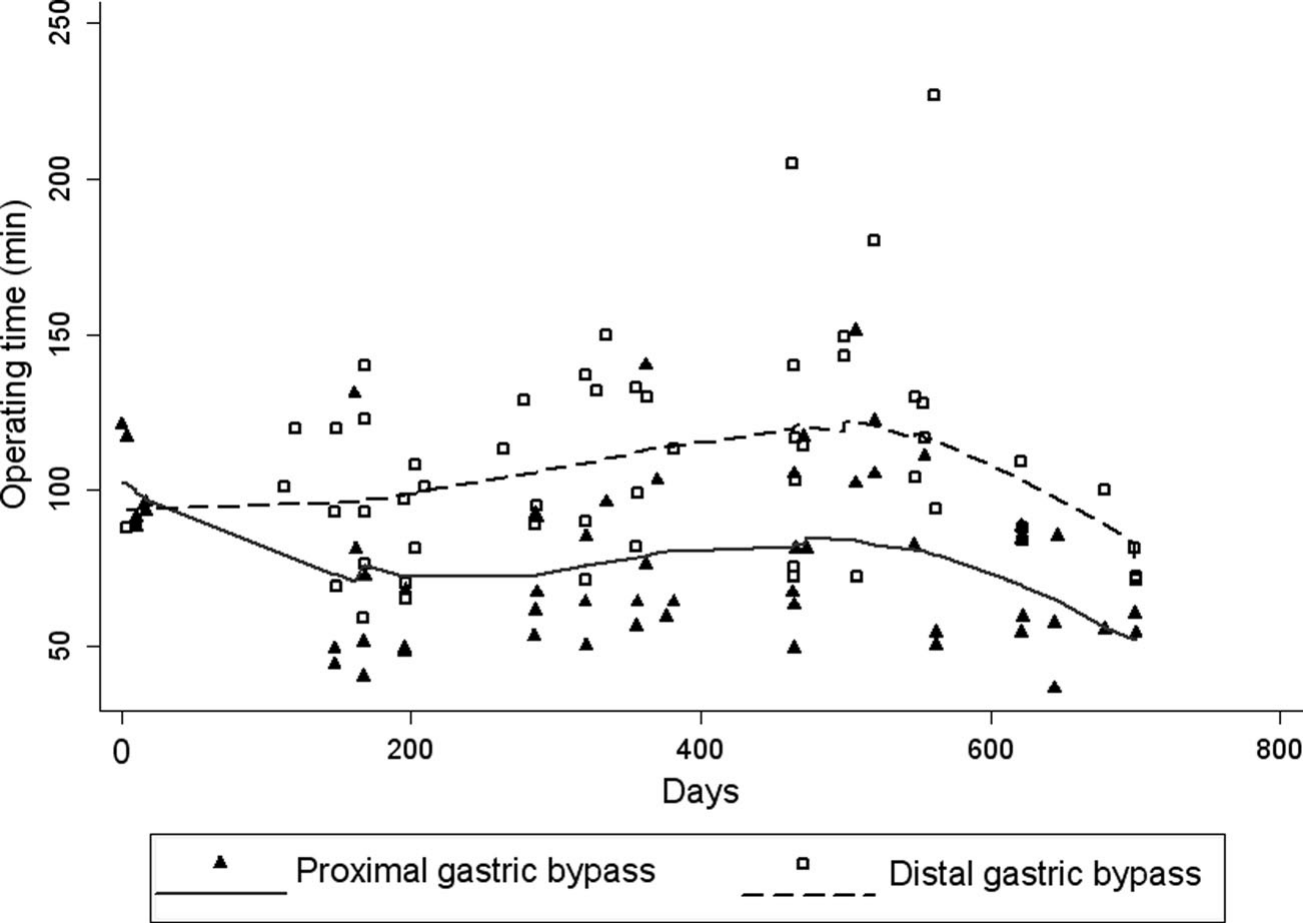
Operating times for proximal (*triangle*) and distal gastric bypass (*squares*) over the course of the study. Days from study start are indicated on the *x-axis*, and operating times in minutes on the *y-axis*. The corresponding fitted curves are estimated by LOWESS-regression curves (locally weighted scatterplot smoothing)

Baseline patient characteristics are described in Table 1. All patients had an American Society of Anesthesiologists physical status classification of 3. The patients lost mean 8.4 kg (SD 5.1) from inclusion to the day of surgery, corresponding to a mean weight loss of 5.3 %, *p* = 0.12 between groups. The median operating time was 72 (36–151) min in the proximal group and 101 (59–227) min in the distal group, *p* < 0.001. The median number of procedures in the study per surgeon was 22 (range 16–29), and 65 % of cases were performed by two senior bariatric surgeons. In both procedures, the median operating times varied between the surgeons. For proximal gastric bypass, the median operating times per surgeon ranged from 50 to 108 min, and for distal gastric bypass from 84 to 123 min. Operating times for each procedure are shown in Fig. 3. Two procedures in the distal group were converted to laparotomy; one due to intraabdominal adhesions and one due to a short bowel mesentery causing technical difficulties. The median lengths of hospital stay after proximal and distal gastric bypass were 2 (1–4) and 2 (1–24) days, respectively, *p* = 0.29. However, no patients in the proximal group stayed longer than 4 days, while seven patients in the distal group stayed longer than 5 days, including two who stayed for more than 20 days.

**Table 1:** Patient characteristics at baseline of 113 patients (BMI 50–60 kg/m^2^) randomized to either proximal or distal gastric bypass

	Proximal gastric bypass (*n* = 57)	Distal gastric bypass (*n* = 56)
Age, year^a^	39.4 (9.3)	42.0 (8.2)
Women^b^	36 (63 %)	37 (66 %)
Weight, kg^a^	160 (20)	157 (17)
BMI, kg/m^2a^	53.3 (2.5)	53.6 (3.3)
Systolic BP, mmHg^a^	131 (16)	138 (17)
Diastolic BP, mmHg^a^	80 (11)	80 (12)
Diabetes mellitus type 2^b^	14 (25 %)	19 (34 %)
Hypertension^b^	33 (58 %)	34 (61 %)
OSA^b^	21 (36 %)	19 (34 %)
CPAP-dependent OSA^b^	17 (30 %)	14 (25 %)
Joint pain^b^	33 (58 %)	40 (71 %)
Depression^b^	13 (23 %)	9 (16 %)
Urinary incontinence^b^	10 (18 %)	13 (23 %)
Gastroesopheagal reflux^b^	14 (25 %)	16 (29 %)
Hypothyroidism^b^	3 (5 %)	11 (20 %)
Current smoker^b^	8 (14 %)	14 (25 %)

^a^Mean (SD)

^b^Number of patients (percentage)

*BMI* body mass index, *CPAP* continuous positive airway pressure, *OSA* obstructive sleep apnea, *BP* blood pressure

Complications categorized according to the Contracted Accordion Classification are listed in Table 2. The number of patients with complications (5 vs 10, *p* = 0.18) and the distribution of complications categorized according to the Contracted Accordion Classification (*p* = 0.11) did not differ significantly. All patients with severe (grade 3) complications, all resulting in reoperation, had received a distal gastric bypass (0 vs 6, *p* = 0.01). There were no deaths.

**Table 2:** Perioperative complications in superobese patients randomized to either proximal or distal gastric bypass stratified according to the Contracted Accordion Classification

Complications	Proximal gastric bypass (*n* = 57)	Distal gastric bypass (*n* = 56)
Patients with no complication	52 (91 %)	46 (82 %)
1. Mild complications	4	2
Pneumomediastinum	1	0
Hematoma	1	1
Hematochezia	0	1
Superficial skin burn from warm liver retractor	1	0
Hypertension	1	0
2. Moderate complications	1	2
Urinary tract infection	1	0
Intraabdominal abscess	0	1
Melena	0	1
3. Severe complications	0	6
Small bowel obstruction	0	2
Intraabdominal bleeding	0	1
Leakage at the enteroenteroanastomosis	0	1
Small bowel perforation	0	1^a^
Ventral hernia recurrence	0	1
4. Deaths	0	0

^a^The patient underwent a second laparotomy due to bleeding after removal of an abdominal drain

Six patients, all in the distal gastric bypass group, underwent a total of seven reoperations. Three of the reoperations were completed laparoscopically and were the result of staple line bleeding, internal herniation, and small bowel entrapment under an intraperitoneal onlay mesh. Three reoperations were performed by laparotomy. One was the result of a leakage from the enteroenterostomy and one the obstruction of the enteroenteroanastomosis, with both requiring a new anastomosis. The third patient had an iatrogenic small bowel injury requiring suture, and had a second laparotomy due to bleeding after the removal of a non-suction abdominal drain.

## Discussion

In the present double-blind, randomized controlled trial, we found that in patients with a BMI of 50 to 60 kg/m^2^, distal gastric bypass was associated with longer operating time than proximal gastric bypass. The overall frequency of complications did not differ; however, distal gastric bypass resulted in more severe postoperative complications requiring reoperation than proximal gastric bypass.

The overall complication rate in the proximal group of 9.4 % is comparable with that found in other studies of patients with a BMI ≥ 50 kg/m^2^ [6, 18]. Our finding of 18.5 % overall complication rate in the distal group is higher than reported in two case series of distal gastric bypass reporting 2.8 % (laparoscopic) and 13 % (open) major complications. These studies, however, reported only major morbidity [13, 19]. In contrast, one non-randomized controlled study of gastric bypass versus distal gastric bypass noted 32 % complications in both groups, with a 6 % overall rate of reoperation [20]. The statistical comparison of complications in the two groups should be interpreted with caution due to the low number of events.

To the best of our knowledge, five randomized controlled trials have compared intestinal limb lengths in bariatric surgery [21–25], of which four included superobese patients. Four of eight retrospective studies that evaluated the outcome of varying limb lengths included superobese patients [5, 11, 26, 27]. Few of the studies reported on perioperative outcome, and none have used standardized classification of complications, making direct comparison of outcomes difficult. Our finding of longer operating times when performing distal gastric bypass has been described by others and may reflect technical surgical aspects [20]. The main difference between the proximal and distal bypass is the handling of the small bowel. There is more manipulation, and the long alimentary limb increases the strain on the enteroenteroanastomosis. Most reoperations observed in our study were related to the small bowel. Changes in surgical technique and greater experience with the handling of the distal small bowel while creating the enteroenteroanastomosis might reduce the differences in operating time and complication rates. Dividing the short bowel before fashioning the gastrojejunal or the jejunojejunal anastomosis might lead to less small bowel-related complications than using the omega loop technique. Of the other studies, only Nergaard et al. describes using the omega loop technique [25], but in most studies this is not specified. Some advocate dividing the mesenterium beyond the first vascular arcade to reduce the strain on the anastomosis, but we have not used this method routinely, and increased strain on the jejunoileal anastomosis was not perceived to be a problem during the procedures.

The incidence of short-term complications for biliopancreatic diversion with duodenal switch, an alternative malabsorptive procedure, is also high. Two previous studies of duodenal switch in superobese patients, of which one was from our study group, both present complications in 24 % of the patients within 30 days of surgery, one reporting 12 % severe complications [28, 29]. In a recent single-center review, laparoscopic duodenal switch had a significantly higher leak rate of 5 % compared to proximal gastric bypass [8]. The distal gastric bypass may be an alternative to duodenal switch in superobese patients, although there is little evidence as to long-term outcome. Malabsorptive bariatric procedures, however, can impose adverse events that may offset the benefits of increased weight loss in the long term [30].

The major strengths of the present study include the double-blind, randomized controlled design, a high follow-up rate, and standardization of the surgical procedures. In addition, we used a well-established grading system for reporting complications, which is essential for facilitation of comparison with other studies. The study was not powered for analyzing differences in complications, thus limiting reasonable statistical comparisons of outcome between the two groups. We restricted the study to patients with a BMI of 50 to 60 kg/m^2^, and therefore the results may not apply to patients with a BMI outside of this range. The surgeons had greater experience performing proximal gastric bypass as opposed to distal gastric bypass, and learning curve effects may thus contribute to the differences observed in postoperative outcome. We tried to minimize this potential bias by limiting the number of surgeons involved; however, the small number of procedures suggest that the surgeons were still in the learning curve for the technical steps that differ between proximal and distal gastric bypass. However, the procedures are identical in all other parts than the handling of the small bowel.

The technical skills of the individual surgeon are likely to be of importance for the risk of complications. Birkmeyer et al. have shown that technical skills correlate with less complications and shorter operating time [31]. The small number of patients in our study does not allow for reasonable statistical evaluation of any potential correlation between operating times and complications; however, the mean operating times are shorter than in comparable studies [19, 20]. Cook et al. found that by standardizing the anesthetic agents and the operative steps, length of hospital stay and outcomes in regard to cardiac surgical practice improved [32]. To facilitate the use of standardized surgical technique in the present study, the surgeons at the two study centers operated a few patients together before the outset of the study. Detailed description of operative techniques might allow for more standardized care for bariatric patients and may enable comparison between studies.

Measuring the small bowel by using graspers or umbilical tape introduces risks of measurement biases. We are not aware of any studies validating these methods. Measurement of the total bowel length could have given more information on procedural effect on postoperative nutritional status and weight loss.

## Conclusion

Distal gastric bypass was associated with longer operating time and a higher frequency of reoperations and severe perioperative complications than proximal gastric bypass surgery in patients with BMI 50–60 kg/m^2^.
